# Innovative Synoptic Reporting With Seven-Point Sampling Protocol to Improve Detection Rate of Microvascular Invasion in Hepatocellular Carcinoma

**DOI:** 10.3389/fonc.2021.726239

**Published:** 2021-11-04

**Authors:** Bing Liao, Lijuan Liu, Lihong Wei, Yuefeng Wang, Lili Chen, Qinghua Cao, Qian Zhou, Han Xiao, Shuling Chen, Sui Peng, Shaoqiang Li, Ming Kuang

**Affiliations:** ^1^ Department of Pathology, The First Affiliated Hospital of Sun Yat-sen University, Guangzhou, China; ^2^ Department of Liver Surgery, The First Affiliated Hospital of Sun Yat-sen University, Guangzhou, China; ^3^ Clinical Trials Unit, The First Affiliated Hospital of Sun Yat-sen University, Guangzhou, China; ^4^ Division of Interventional Ultrasound, The First Affiliated Hospital of Sun Yat-sen University, Guangzhou, China; ^5^ Institute of Precision Medicine, The First Affiliated Hospital of Sun Yat-sen University, Guangzhou, China; ^6^ Department of Gastroenterology and Hepatology, The First Affiliated Hospital of Sun Yat-sen University, Guangzhou, China

**Keywords:** hepatocellular carcinoma, microvascular invasion, innovative synoptic reporting, seven-point sampling, SPRING protocol

## Abstract

Pathological MVI diagnosis could help to determine the prognosis and need for adjuvant therapy in hepatocellular carcinoma (HCC). However, narrative reporting (NR) would miss relevant clinical information and non-standardized sampling would underestimate MVI detection. Our objective was to explore the impact of innovative synoptic reporting (SR) and seven-point sampling (SPRING) protocol on microvascular invasion (MVI) rate and patient outcomes. In retrospective cohort, we extracted MVI status from NR in three centers and re-reviewed specimen sections by SR recommended by the College of American Pathologists (CAP) in our center. In prospective cohort, our center implemented the SPRING protocol, and external centers remained traditional pathological examination. MVI rate was compared between our center and external centers in both cohorts. Recurrence-free survival (RFS) before and after implementation was calculated by Kaplan-Meier method and compared by the log-rank test. In retrospective study, we found there was no significant difference in MVI rate between our center and external centers [10.3% (115/1112) *vs.* 12.4% (35/282), P=0.316]. In our center, SR recommended by CAP improved the MVI detection rate from 10.3 to 38.6% (P<0.001). In prospective study, the MVI rate in our center under SPRING was significantly higher than external centers (53.2 *vs.* 17%, P<0.001). RFS of MVI (−) patients improved after SPRING in our center (P=0.010), but it remained unchanged in MVI (+) patients (P=0.200). We conclude that the SR recommended by CAP could help to improve MVI detection rate. Our SPRING protocol could help to further improve the MVI rate and optimize prognostic stratification for HCC patients.

## Introduction

The incidence of hepatocellular carcinoma (HCC) is rising globally. China contributes almost half of new-diagnosed HCC cases in the world, and HCC ranks the second in malignancy mortality in this country (1). Microvascular invasion (MVI) refers to the microscopic finding of cancer cell nest within vessels lined by endothelium (2). It frequently occurs in HCC and is significantly associated with early recurrence and poor survival outcomes of HCC patients (3). Currently, many studies have indicated adjuvant transarterial chemotherapy after hepatic resection could help to improve long-term survival in MVI-positive patients (4–8). However, previous studies showed the MVI positive rate after hepatectomy in pathology report varied substantially, from 7.8 to 57.1% (9). Thus, an accurate and standardized report of MVI is needed for precise patient stratification and consequent individualized treatments.

Traditional pathological narrative report (NR) is no longer considered adequate to report relevant clinical information as it is a paragraph that mainly describes morphological features of tumors (10, 11). In contrast, synoptic report (SR) that includes mandatory parameters in a standardized structure is found effective to improve completeness and accuracy in surgical pathology (12–14). The College of American Pathologists (CAP) published and regularly updated templates of SRs covering a wide range of cancer types that forms the basis of SRs produced in clinical practice (15, 16). For instance, pathological studies on colorectal cancer (CRC) have proven that its high-risk features of recurrence including extramural vascular invasion (EMVI), lymph-vascular invasion (LVI), and perineural invasion that were under-reported in NR increased significantly in SR. Based on the SR, more adjuvant therapies were delivered and better patient outcomes were achieved (17). In pancreatic cancer, SR led to substantially higher detection rates of adverse prognostic factors including resection margin involvement and regional lymph node metastasis, thereby yielding a better overall survival compared to NR (18). However, whether SR recommended by CAP could also improve the detection rate of MVI in HCC has not yet been explored.

Standardized tissue sampling method is essential for the quality of pathology reports and consequent diagnosis (19). Traditionally, HCC sampling focuses on confirmation of the histological features of HCC, completeness of surgical excision, and cirrhosis condition (20, 21). Given that MVI is unevenly distributed in the adjacent liver parenchyma around HCC (2), traditional sampling method usually resulted in false-negative detection (9). The MVI rate under traditional sampling was reported varied from 7.8 to 28.4% (22, 23). A seven-point sampling protocol in the resected liver specimens for MVI detection was proposed by a Chinese consensus (2, 24, 25), which could increase the MVI detection rate to be around 50% in both Eastern Hepatobiliary Surgery Hospital (26) and our center (9, 24). It seems that such a seven-point sampling protocol should be recommended for detection of MVI after HCC resection. However, it has not been applied widely in the country, majorly because of lacking research evidence.

Combination of SR and seven-point sampling protocol may significantly increase the detection rate of MVI in HCC patients. To test the impact of Innovative SR with Seven-Point Sampling (SPRING) protocol on MVI detection rate and patient outcomes, we performed a large population-based multicentric cohort study.

## Materials and Methods

### Patient Population

**Figure 1:**
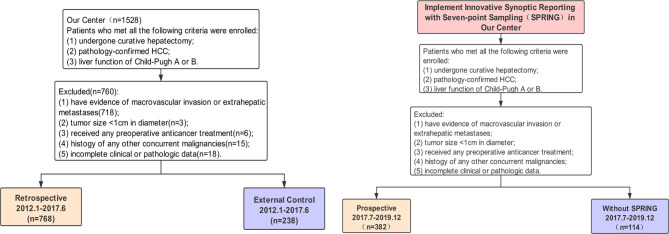
Inclusion and exclusion diagram.

Patients with HCC who underwent curative liver resection were included retrospectively between January 1, 2012, and March 31, 2017 (retrospective cohort), and were prospectively enrolled between April 1, 2017, and December 31, 2019 (prospective cohort), from three tertiary medical centers. The inclusion criteria were as follows: (1) pathologically confirmed HCC; (2) received curative hepatectomy as the initial treatment; (3) liver function of Child-Turcotte-Pugh class A or B; (4) no evidence of macrovascular invasion or extrahepatic metastases. Patients who met any of the following criteria were excluded: (1) received any preoperative anticancer therapy; (2) tumor size <1 cm in diameter; (3) history of any other concurrent malignancies; (4) incomplete clinical or pathological data. A total of 1,180 eligible patients in retrospective cohort and 557 in prospective cohort were enrolled in this study as shown in **Figure 1**. Our study was approved by the ethics committees of all three centers and was in full accordance with the guidelines of the Declaration of Helsinki. Informed consent was obtained from all patients in the prospective study (No. [2018] 072) and waived in the retrospective study. Patients’ demographic data, preoperative laboratory tests, imaging examination, histopathology, and oncological outcomes were extracted from electronic clinical archives. MVI is defined as the presence of tumor cell clusters within the vascular space of the surrounding liver tissue, which is lined by endothelium and visible only under the microscope (2). Tumor status was evaluated every 3 months for the first 2 years and every 6 months since the third year. Observed endpoints included MVI detection rate (defined as the proportion of MVI-positive patients to total patients who received curative resection per year), and recurrent-free survival (RFS, defined as the time interval between the date of HCC diagnosis and the date of tumor recurrence or death).

### NR and Innovative Synoptic Reporting

**Figure 2:**
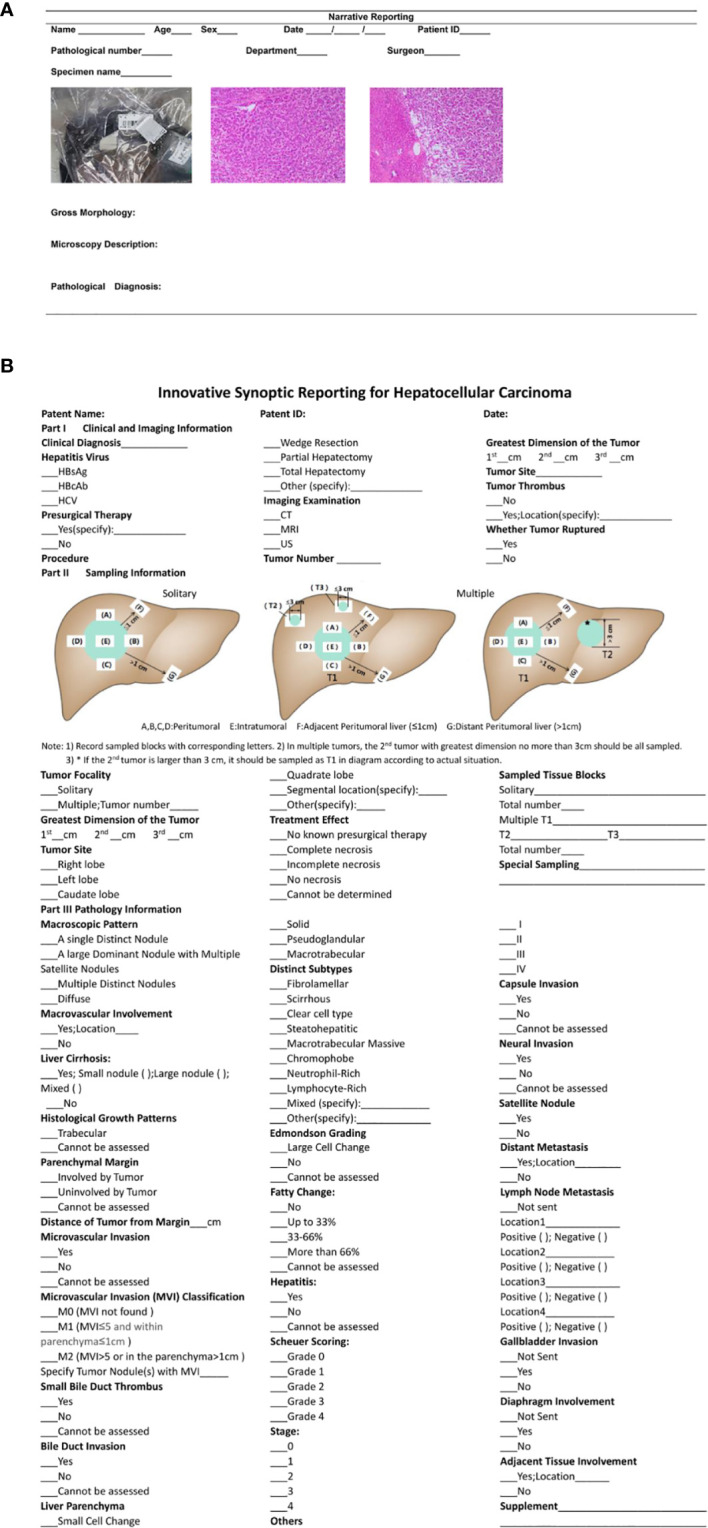
**(A)** Traditional Narrative Reporting (NR); **(B)** Innovative Synoptic Reporting (SR-hcc).

Traditionally, NR includes the following three parts: macroscopy, microscopy, and conclusion with free text (**Figure 2A**). In contrast, a template of SR recommended by CAP outlined the required data elements in HCC pathology (27). Innovatively, we merged clinical and imaging and sampling information in our SR and named it SR-hcc (**Figure 2B**). Pertinent clinical information included clinical diagnosis, hepatitis virus, presurgical therapy, and type of surgical procedure. Imaging information included the type of examination, tumor size, tumor number, tumor site, whether tumor thrombus, and whether ruptured. The diagram of seven-point sampling was displayed, and information on tumor focality, sampled tissue blocks, and total sampling number was listed. Pathologists would check whether the sampling location was appropriate and whether the sampling number was sufficient (generally no less than seven). Once unqualified sampling was found, resampling would be performed within 1 week when surgical specimens were available. Detailed parameters on the pathology part are shown in **Figure 2B**.

### Traditional Sampling and Seven-Point Sampling

Traditionally, HCC specimens are sampled according to Rosai and Ackerman’s Surgical Pathology. It requires one tissue block sampled from the tumor area, transition areas (across tumor and adjacent liver tissues), and proximal liver parenchyma, respectively (28).

In seven-point sampling procedure, all the specimens were cut apart along the maximal tumor section and then were sliced into serial 1 cm thick sections parallel to the maximal tumor section. The solitary tumor should be sampled at least four sites in the peritumoral area (at the junction of the tumor and adjacent liver tissue in a 1:1 ratio at the 12, 3, 6, and 9 o’clock positions), one site in the tumor area (more sites should be sampled for tumors harboring different textures or colors), and one site each in proximal (≦1.0 cm from the tumor) and distal (>1.0 cm from the tumor) paracancerous liver parenchyma if applicable (2). In the case of multiple tumors, the largest dominant nodule should be sampled as described above. If the maximum diameter of the 2^nd^ nodule does not exceed 3 cm, it should be all sampled in one block. Otherwise, it should be sampled as seven-point sampling protocol according to the actual situation. The sampling procedure should be completed within 30 min after surgical removal of specimen for sectioning and fixation (2).

### Spring

Our SR-hcc with sven-point Sampling constituted the SPRING protocol. For each patient, surgeons would fill in the clinical and imaging part in the SR-hcc and submit it with the resected specimen. Then specific sampling pathologists would sample according to seven-point sampling protocol, mark sampled tissue blocks in the sampling diagram, count the total sampling number, and finish the sampling. Four senior pathologists would evaluate the sections and finally finish pathology parts in SR-hcc. The diagnosis on MVI was based on peritumoral samples.

### Retrospective Study

To evaluate whether the use of SR recommended by CAP could increase the detected rate of MVI, we retrospectively included a total of 1,006 HCC cases from three Chinese medical centers between January 1, 2012, and March 31, 2017, including 768 cases from the First Affiliated Hospital of Sun Yat-sen University as the primary group, 238 cases from the Zhujiang Hospital of Southern Medical University and Dongguan People’s Hospital as the external group (**Figure 1**). Sampling procedure was performed in traditional pattern, and MVI information was obtained from the original pathologic reports yielded by NR in both groups. Two senior pathologists retrospectively re-reviewed specimen sections using SR recommended by CAP (21) (SR-CAP) in the primary group but did not in the external group. The MVI detection rate was compared between NR and SR-CAP.

### Prospective Study

SR-hcc was applied in the First Affiliated Hospital of Sun Yat-sen University since April 1, 2017. We prospectively enrolled a total of 382 patients from April 1, 2017, to December 31, 2019, with SR-hcc implementation. In the same period, 114 patients from two external centers were also prospectively recruited, and their pathological reports remained using NR and specimen sampling remained the traditional pattern. The MVI detection rates were then compared between our center and external centers in this prospective cohort.

### Trends of MVI Rate Under Interrupted Time Series Design

Next, observed trend in MVI detection rate following the implementation of SPRING (the “interruption”) protocol was compared with trend in the absence of the protocol. An interrupted time series (ITS) design (29) was conducted every 6 months before (January 1, 2012, to March 31, 2017) and after (July 1, 2017, to December 31, 2019) application of SPRING. To account for 3-month probation for implementation, during which the data were not stable, we excluded the 3-month (April 1, 2017, to June 30, 2017) following implementation in ITS analysis. This allowed for post-interruption trends to better coincide with the actual impact of the protocol.

### Statistical Analysis

Descriptive statistics were used to tabulate patient characteristics. Continuous variables were shown as means ± standard deviation and categorical variables as numbers and percentages. Differences between the primary group and external group in the retrospective cohort and differences between our center and external center in the prospective cohort were assessed using the t-test, chi-square test, and Wilcoxon signed-rank test. The Kaplan-Meier survival curves of RFS pre- and post-implementation were plotted and compared by the log-rank test in our center.

Differences in MVI detection rate and sampling number between pre- and post-implementation periods were assessed using segmented regression through ITS analysis. Separate models were fit to primary cohort and external controls. Models were tested for overdispersion and autocorrelation using recommended methods. Results were reported as average incidence rate and 95% CIs. All statistical tests were two-sided, with *P<0.050* considered statistically significant. All analyses were conducted on SAS 9.5 and R 3.6.1.

## Results

### Baseline Characteristics

In the retrospective cohort (n=1,006, **Table 1**), fewer patients with BCLC 0-A tumors (81.1 *vs.* 87.0%, *P=0.038*), fewer patients with tumor size ≥5 cm (47.8 *vs.* 55.5%, *P=0.038*), and more patients with multifocal tumors (21.4 *vs.* 13.9%, *P=0.021*) were found in the primary group compared to the external group. Other variables were comparable between the two groups (all *P>0.050*).

**Table 1:** Baseline characters for all patients in our center and external centers.

Variables	Levels	Retrospective cohort (201201–201706)				Prospective cohort (201707–201912)			
		Total	Our center	External centers	Pvalue^1^	Total	Our center	External centers	Pvalue^1^
Age	Mean (SD)	53.9 (11.7)	53.9 (11.7)	54.0 (11.4)	0.990^#^	54.4 (11.4)	53.7 (11.1)	56.5 (12.3)	0.025
(yr)	Median (IQR)	54.8 (45.4,62.0)	54.7 (45.5,62.1)	55.0 (45.4,61.8)		54.3 (46.3,62.8)	53.4 (46.2,62.1)	57.1 (46.6,65.7)	
Gender	Male	874 (86.9%)	663 (86.3%)	211 (88.0%)	0.353	434 (87.5%)	335 (87.7%)	99 (86.8%)	0.809
	Female	132 (13.1%)	105 (13.7%)	27 (11.3%)		62 (12.5%)	47 (12.3%)	15 (13.2%)	
HBsAg	Negative	144 (14.3%)	113 (14.7%)	31 (13.0%)	0.516	92 (18.5%)	62 (16.2%)	30 (26.3%)	0.015
	Positive	862 (85.7%)	655 (85.3%)	207 (87.0%)		404 (81.5%)	320 (83.8%)	84 (73.7%)	
HCV	Negative	984 (97.8%)	750 (97.7%)	234 (98.3%)	0.541	480 (96.8%)	367 (96.1%)	113 (99.1%)	0.136
	Positive	22 (2.2%)	18 (2.3%)	4 (1.7%)		16 (3.2%)	15 (3.9%)	1 (0.9%)	
PLT	<100	111 (11.0%)	78 (10.2%)	33 (13.9%)	0.111	37 (7.5%)	26 (6.8%)	11 (9.6%)	0.311
(×10^9^/L)	≥100	895 (89.0%)	690 (89.8%)	205 (86.1%)		459 (92.5%)	356 (93.2%)	103 (90.4%)	
AFP	Mean (SD)	15183 (101E3)	15838 (111E3)	13069 (54884)	0.499^#^	15408 (94567)	18224 (107E3)	5972 (24373)	0.247^#^
Level (ng/ml)	Median (IQR)	46.0 (5.6,842.8)	53.9 (5.9,756.1)	31.8 (4.8,1092)		35.2 (5.0,639.1)	25.9 (4.9,577.7)	65.5 (5.2,1197)	
AFP	≤20	411 (40.9%)	304 (39.6%)	107 (45.0%)	0.222	224 (45.2%)	179 (46.9%)	45 (39.5%)	0.310
Group	20–400	285 (28.3%)	227 (29.6%)	58 (24.4%)		127 (25.6%)	97 (25.4%)	30 (26.3%)	
(ng/ml)	≥400	310 (30.8%)	237 (30.9%)	73 (30.7%)		145 (29.2%)	106 (27.7%)	39 (34.2%)	
Tumor	Mean (SD)	5.7 (3.2)	5.6 (3.1)	5.9 (3.7)	0.573^#^	6.0 (6.2)	6.1 (6.8)	5.7 (3.3)	0.802^#^
Size (cm)	Median (IQR)	4.9 (3.4,7.3)	4.8 (3.4,7.3)	5.2 (3.3,7.0)		4.9 (3.3,7.1)	4.8 (3.3,7.1)	5.2 (3.3,7.1)	
Tumor	1–3 cm	187 (18.6%)	141 (18.4%)	46 (19.3%)	0.038	106 (21.4%)	81 (21.2%)	25 (21.9%)	0.765
Size	3–5 cm	320 (31.8%)	260 (33.9%)	60 (25.2%)		144 (29.0%)	114 (29.8%)	30 (26.3%)	
Group	≥5 cm	499 (49.6%)	367 (47.8%)	132 (55.5%)		246 (49.6%)	187 (49.0%)	59 (51.8%)	
Tumor	1	809 (80.4%)	604 (78.6%)	205 (86.1%)	0.021	410 (82.7%)	304 (79.6%)	106 (93.0%)	0.009
Number	2	102 (10.1%)	87 (11.3%)	15 (6.3%)		51 (10.3%)	45 (11.8%)	6 (5.3%)	
Group	3	27 (2.7%)	25 (3.3%)	2 (0.8%)		10 (2.0%)	10 (2.6%)	0 (0.0%)	
	>3	68 (6.8%)	52 (6.8%)	16 (6.7%)		25 (5.0%)	23 (6.0%)	2 (1.8%)	
BCLC	0A	830 (82.5%)	623 (81.1%)	207 (87.0%)	0.038	420 (84.7%)	314 (82.2%)	106 (93.0%)	0.005
Group	B	176 (17.5%)	145 (18.9%)	31 (13.0%)		76 (15.3%)	68 (17.8%)	8 (7.0%)	
MVI	MVI−	909 (90.4%)	698 (90.9%)	211 (88.7%)	0.309	290 (58.5%)	192 (50.3%)	98 (86.0%)	<0.001
Status	MVI+	97 (9.6%)	70 (9.1%)	27 (11.3%)		206 (41.5%)	190 (49.7%)	16 (14.0%)	

PLT, platelet; AFP, alpha fetoprotein; MVI, microvascular invasion; SD, standard deviation; IQR, interquartile range. ^1^Principles for selecting P values and statistics: (1) For continuous variables, if they met normal distribution, we used T-test results; otherwise, we used Wilcoxon results (“#” means that continuous variables did not meet normal distribution). (2) For categorical variables, we used chi-square test or Fisher exact probability method. 2. Data description method: (1) For continuous variables, if they satisfied normal distribution, we selected the mean (SD); otherwise, we selected the median (IQR). (2) For categorical variables, they were described as N (%) under different categories.

In the prospective cohort (n=496, **Table 1**), fewer patients with BCLC 0-A tumors (82.2 *vs.* 93.0%, *P=0.005*), more patients with multifocal tumors (20.4 *vs.* 7.0%, *P=0.009*), more patients with positive HBsAg (83.8 *vs.* 73.7%, *P=0.015*), and a younger average age [53.7 (11.1) *vs.* 56.5 (12.3), *P=0.025*] were found in our center compared to the external center. Other variables were comparable among three centers (all *P>0.050*). The comparisons of clinicopathological characteristics between MVI-positive and MVI-negative groups after SPRING in our center and external center are displayed in **Supplementary Tables 1** and **2** (see Table, Supplemental Digital Content, which demonstrated patients’ clinicopathological characteristics), respectively.

We also compared baseline characters between retrospective and prospective cohort in our center and external centers in **Supplementary Tables 3** and **4** (see Table, **Supplemental Digital Content**, which demonstrated patients’ baseline characteristics). The comparison of baseline characters between our center and external centers throughout the whole study period is shown in **Supplementary Table 5** (see Table, **Supplemental Digital Content**, which demonstrated all patients’ baseline characteristics).

### The MVI Detection Rate in the Retrospective Study Under SR-CAP

**Figure 3:**
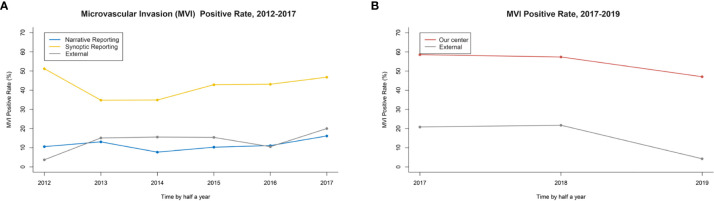
**(A)** The MVI rate in retrospective study: Synoptic Reporting recommended by the College of American Pathologists (SR-CAP) in the primary group *vs.* NR in the primary and external group. **(B)** The MVI rate in the prospective study: Our Center *vs.* External Center.

In the retrospective cohort, the overall MVI detection rate of three centers was 9.6% (97/1,006). Regarding MVI rate reported by original NR, there was no significant difference between the primary group and the external group (9.1 *vs.* 11.3%, *P=0.309*) (**Table 1**). After re-reviewing specimen sections using SR recommended by CAP in the primary group, the MVI detection rate increased significantly compared to it reported previously by NR (38.7 *vs.* 9.1%, *P<0.001*). Also, the MVI detection rate in the primary group reported by SR-CAP was significantly higher than that in the external group reported by NR (38.7 *vs.* 11.3%, *P<0.001*) (**Figure 3A**).

### The MVI Detection Rate in the Prospective Study Under SPRING

After April 1, 2017, the SPRING protocol was implemented in our center. The MVI detection rate by SPRING in our center was significantly higher than that by traditional pathology examination in the external center (49.7 *vs.* 14.0%, *P<0.001*) (**Figure 3B**).

**Supplementary Tables 6** and **7** (see Table, **Supplemental Digital Content**, which demonstrated the comparison of baseline characteristics between 2018 and 2019 in our center and external centers, respectively) showed the MVI rate declined in 2019 compared to 2018 (our center: 53.7 *vs.* 45.1%, *P=0.120*; external centers: 22.2 *vs.* 4.3%, *P=0.012*). The tumor size was smaller in 2019 in our center [6.8 (9.0) cm *vs.* 5.5 (5.1) cm, *P=0.001*], and more unifocal patients were in 2019 in external centers [Tumor number group 1: 38 (84.4%) *vs.* 45 (97.8%), *P=0.045*].

### Subgroup Analysis in the Prospective Study

Subgroup analysis was performed according to influencing factors of MVI rate including tumor size, tumor number, BCLC stage, and alpha fetoprotein (AFP) level (9) (**Table 2**). The comparison of MVI rate between our center with SPRING and external center with traditional protocol was 30.9 *vs.* 8.0% (*P=0.022*), 38.6 *vs.* 3.3% (*P<0.001*), and 64.7 *vs.* 22.0% (*P<0.001*) in 1.0–3.0, 3.0–5.0 cm, and ≧5.0 cm group, respectively. Concerning the tumor number, in the single tumor group, MVI rate in our center was significantly higher than that in the external center (47.4 *vs.* 12.3%, *P<0.001*), but this advantage was not significant in multifocal groups (two tumors group: 57.8 *vs.* 33.3%, *P=0.390*; three tumors group: 60.0% *vs.* 0, *P=NA*, >3 tumors group: 60.9 *vs.* 50.0%, *P=1.000*). As for BCLC stage, the MVI rate of 0A-stage patients in our center was higher than that in the external center (47.8 *vs.* 12.3%, *P<0.001*), and the MVI rate was comparable in B-stage patients (58.8 *vs.* 37.5%, *P=0.283*). All subgroups related to AFP level showed an improved MVI rate in our center, and detailed data are shown in **Table 2**. We noticed the improvement of MVI detection under SPRING was more significant in tumor size ≧5.0 cm group and AFP ≧400 group (67.9 *vs.* 20.5%, *P<0.001*).

**Table 2:** Subgroup analysis for prospective cohort (our center *vs.* external centers).

	Variables	Levels	Total	Our center	External centers	Pvalue^1^
	Overall	MVI−	290 (58.5%)	192 (50.3%)	98 (86.0%)	<0.001
		MVI+	206 (41.5%)	190 (49.7%)	16 (14.0%)	
Tumor Size	1.0–3.0 cm	MVI−	79 (74.5%)	56 (69.1%)	23 (92.0%)	0.022
		MVI+	27 (25.5%)	25 (30.9%)	2 (8.0%)	
	3.0–5.0 cm	MVI−	99 (68.8%)	70 (61.4%)	29 (96.7%)	<0.001
		MVI+	45 (31.3%)	44 (38.6%)	1 (3.3%)	
	≥5.0 cm	MVI−	112 (45.5%)	66 (35.3%)	46 (78.0%)	<0.001
		MVI+	134 (54.5%)	121 (64.7%)	13 (22.0%)	
Tumor Number	1	MVI−	253 (61.7%)	160 (52.6%)	93 (87.7%)	<0.001
		MVI+	157 (38.3%)	144 (47.4%)	13 (12.3%)	
	2	MVI−	23 (45.1%)	19 (42.2%)	4 (66.7%)	0.390
		MVI+	28 (54.9%)	26 (57.8%)	2 (33.3%)	
	3	MVI−	4 (40.0%)	4 (40.0%)	0	Not Applicable
		MVI+	6 (60.0%)	6 (60.0%)	0	
	>3	MVI−	10 (40.0%)	9 (39.1%)	1 (50.0%)	1.000
		MVI+	15 (60.0%)	14 (60.9%)	1 (50.0%)	
BCLC Staging	0A	MVI−	257 (61.2%)	164 (52.2%)	93 (87.7%)	<0.001
		MVI+	163 (38.8%)	150 (47.8%)	13 (12.3%)	
	B	MVI−	33 (43.4%)	28 (41.2%)	5 (62.5%)	0.283
		MVI+	43 (56.6%)	40 (58.8%)	3 (37.5%)	
AFP Level	≦20	MVI−	156 (69.6%)	114 (63.7%)	42 (93.3%)	<0.001
		MVI+	68 (30.4%)	65 (36.3%)	3 (6.7%)	
	20–400	MVI−	69 (54.3%)	44 (45.4%)	25 (83.3%)	<0.001
		MVI+	58 (45.7%)	53 (54.6%)	5 (16.7%)	
	≧400	MVI−	65 (44.8%)	34 (32.1%)	31 (79.5%)	<0.001
		MVI+	80 (55.2%)	72 (67.9%)	8 (20.5%)	

MVI, microvascular invasion; AFP, alpha fetoprotein. ^1^principles for selecting P values and statistics: (1) For continuous variables, if they met normal distribution, we used T-test results; otherwise, we used Wilcoxon results. (2) For categorical variables, we used chi-square test or Fisher exact probability method. 2. Data description method: (1) For continuous variables, if they satisfied normal distribution, we selected the mean (standard deviation); otherwise, we selected the median (interquartile range). (2) For categorical variables, they were described as N (%) under different categories.

### Trends of Sampling Number and MVI Detection Rate Following SPRING

**Figure 4:**
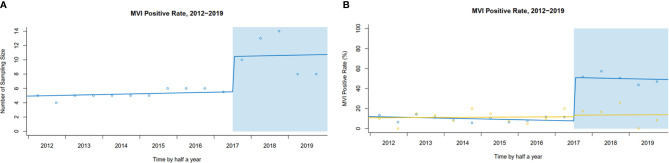
**(A)** Interrupted time series plot of sampling number pre- and post-implementation in our center. **(B)** The trends of MVI rate pre- and post-implementation (Our center *vs.* External center).

In our center, after implementing SPRING, more tissue blocks in peritumoral areas were sampled [median of sampling number: pre 5 (95% CI: 4–6) *vs.* post 10 (95% CI: 8–14), level change 4.9 (95% CI: 2.0–7.8), *P<0.001*] (**Figure 4A**). Following implementation of SPRING, a dramatic increase in MVI detection was found [pre- 9.1% to post- 49.7%; level change: 43.3% (95% CI: 36.4–50.3%), *P<0.001*]. In comparison, a smaller but not statistically significant increase of MVI rate was observed in the external center, which remained the traditional pathological examination [pre- 11.3% to post- 14.0%; level change: 1.6% (95% CI: −12.0–15.2%), *P=0.897*] (**Figure 4B**).

### Prognostic Value of SPRING

**Figure 5:**
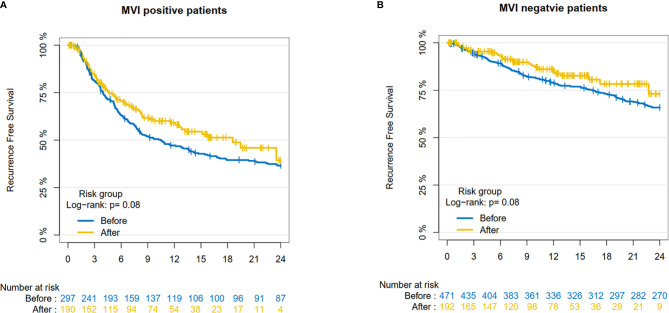
The Recurrence-Free Survival pre- and post-implementation in our center. **(A)** MVI-negative patients; **(B)** MVI-positive patient.

Finally, we investigated the prognostic effect of the SPRING protocol on HCC patients in our center. For MVI-negative patients, 1-year RFS rate was 78.8% (95% CI: 75.1–82.7%) before implementation and 85.0% (95% CI: 79.1–91.3%) after implementation. Two-year RFS rate was 65.9% (95% CI: 61.5–70.5%) before implementation and 73.0% (95% CI: 61.3–87.0%) after implementation. For MVI-positive patients, 1-year RFS rate was 47.2% (95% CI: 41.6–53.5%) before implementation and 59.2% (95% CI: 51.8–67.6%) after implementation. Two-year RFS was 36.6% (95% CI: 31.2–42.9%) before implementation and 39.4% (95% CI: 26.8–57.7%) after implementation. It suggested that the RFS of both MVI-negative patients (*P=0.080*) and MVI-positive patients (*P=0.080*) improved after SPRING, although there was no statistically significant difference (**Figure 5**).

## Discussion

In this study, we demonstrated that SPRING protocol could help improve MVI detection rate and make more accurate risk stratification on patient outcomes, when compared to traditional pathology examination in HCC patients.

Standardized pathology reporting is playing a much more important role in surgical oncology. Studies on pathology reports confirmed that adverse prognostic factors like lymph node and resection margin involvement in pancreatic cancer, as well as EMVI and LVI in CRC were under-reported in traditional NR (17). In our study, an increased MVI rate was reported by SR-CAP in the same patient group (9.1% *vs.* 38.7%, *P<0.001*), indicating MVI in HCC might also be under-reported in traditional NR. MVI was not commonly employed as a routine diagnostic parameter in HCC pathology (25), which might partially cause the neglect of diagnosis of MVI by pathologists. The free-text form of NR could not remind pathologists to report parameters completely in routine diagnosis. In addition, because of different regulations and personal preferences on reporting, inconsistencies of NR were commonly seen among different pathologists and institutions. This non-standardization in NR made it prone to missing information, especially useful parameters for allocating postsurgical adjuvant treatment (11, 30). SR including required pathological parameters could prevent the omission of essential elements (13, 14, 31). Firstly, a structured format prompts pathologists to report the presence or absence of required parameters, probably encouraging a more detailed evaluation under microscopy (31). Secondly, SR possibly urges pathologists to check the diagnostic criteria of parameters in up-to-date guidelines so that they could finish the diagnosis expertly. Finally, after learning guidelines, the growing awareness among pathologists about the effect of poor prognostic factors on disease recurrence and clinical decisions may also attribute to the increased detection rate (32). Our experience in this study shows the application of SR might help to improve pathologists’ awareness in reporting MVI and improve MVI detection rate in HCC.

Currently, the multidisciplinary team (MDT) meetings in oncology were shown to increase the rate of appropriate treatment and improve survival, which needs an adequate exchange of multiple diagnostic information. Meanwhile, pertinent clinical and imaging information would help pathologists narrow the differential diagnosis and improve diagnostic accuracy considering the subjective feature of pathological evaluation (33, 34). However, only 14% of pathology reports were provided with pertinent clinical and imaging data in a French, nationwide survey on hilar cholangiocarcinoma (30). Although the Laboratory Accreditation Program of the CAP has codified pathologists should be fully cognizant of the essential clinical data (34), the appropriate way of providing this information was reported as unavailable (33). Our innovative SR merged clinical and imaging information would better satisfy the need of MDT.

Standardized tissue sampling bases the quality of pathological diagnosis (12, 33). Adequacy assessment was found to improve both sufficiency and quality of specimens, which was widely used in cytopathology like Pap testing in cervical cancer, but rarely used in surgical pathology (35, 36). Whether the sampling location was appropriate and whether the sampling number was sufficient were hard to evaluate because sampling information was rarely reported previously in surgical oncology. Thus, we added detailed sampling diagrams to show concrete sampling location and record total sampling number in SR-hcc. The sampling part in our SR-hcc potentially provided an example for applying adequacy assessment in surgical pathology. Standardization in peritumoral sampling of HCC specimen was usually neglected, which is prone to resulting in negative detection of MVI that is not evenly distributed around the adjacent liver parenchyma. A Chinese consensus recommended seven-point sampling procedure that emphasized adequacy in peritumoral sampling (2) but lacked large-scale clinical evidence. We adopted this seven-point sampling procedure in the SPRING protocol and confirmed an increased MVI detection rate [43.3% (95% CI 36.4–50.3), *P<0.001*].

Most previous studies focused on a certain step in quality improvement and error reduction of pathology diagnosis (33), but they neglected the importance of integral action of the whole process, which has been stressed before (12). Our SPRING protocol, which combined seven-point sampling and SR, helped to improve MVI rate significantly (49.7 *vs.* 14.0%, *P<0.001*). SPRING demonstrated that the standardization from sampling to reporting was effective and could possibly be promoted to many other pathological parameters and cancer types in quality improvement. Moreover, the SPRING protocol facilitated communication and cooperation among surgeons, radiologists, and pathologists, which not only improve the diagnosis quality but also benefit patients in the end.

Subgroup analysis showed the improvement of MVI detection under SPRING was especially pronounced in patients with tumor size ≧5 cm and AFP level ≧400. This may indicate under-reporting of MVI was more common among high-risk patients; thus, pathologists should notice whether their sampling was qualified and perform a more detailed evaluation. As for the grouping of tumor number and BCLC stage, we found that although MVI rate in our center was higher, this advantage was not significant in B-stage and multifocal patients. Small sample size of these groups (B-stage patients: our center n=68, external centers n=8; multifocal patients: our center n=78, external centers n=8) might partly explain the reason. We need to further collect more cases from more centers to validate this improvement in the future. Furthermore, considering for complexity in sampling multifocal specimens, pathologists should pay more attention on the sufficiency of smaller focus in addition to the dominant one.

Many studies examined the quality of pathology diagnosis by comparing the results of reviewing specimen sections by different pathologists, but evaluating the relation of a prognostic factor with the outcome would be more direct and objective (17). For example, after standardization of pathology examination in pancreatic cancer, the overall survival in lymph node involvement–negative patients improved and remained unchanged in positive patients (18). In our study, we explored the prognostic value of MVI status before and after SPRING. The RFS for both MVI-negative patients (*P=0.080*) and MVI-positive patients (*P=0.080*) improved, although the statistical difference was not significant. We speculated that false negative patients reduced after SPRING and more adjuvant therapy was probably applied to MVI-positive patients, which might facilitate the improvement of RFS. SPRING could make it more accurate in risk stratification, thus indirectly reflected the improved quality in pathology evaluation.

There were several limitations to our study. First, we did not re-review the specimen sections from external controls by SR because of unavailable acquisition. Second, our protocol was only implemented in a single center, so further studies are needed to validate its effectiveness in more centers. Third, MVI detection is operator-dependent, so we let the two pathologists who re-reviewed sections in the retrospective study participate in prospective study in our center. We think controlling operator could reduce operator bias, which could better reflect the comparison before and after SPRING in our center. However, as for the comparison between our center and external centers in the prospective study, we needed pathologists who didn’t involve in the retrospective study to be the examiners for pathologists in external centers did not involve in our retrospective study. Unfortunately, it is now hard to distinguish pathology reports made by two pathologists who were involved in retrospective study and the two who did not. During the prospective research period, four pathologists would communicate experience of diagnosis, and there might also be slight influences. We would modify and revise this point in further study. Fourth, in the prospective cohort, the baseline characters of patients in our center and external centers were different in age, HBsAg, tumor number, and BCLC group, so we made a subgroup analysis according to influencing factors of MVI rate including tumor size, tumor number, BCLC stage, and AFP level. We compared MVI rate in each subgroup. The population should be possibly the same in the future study. Our experience in this study may help provide an example for improvement of precise pathological diagnosis with SR and sampling method in surgical oncology.

In conclusion, the SR-hcc and SPRING protocol could help to improve the MVI detection rate in HCC patients who received curative resection, and consequently help decision for potential adjuvant therapy.

## Data Availability Statement

The original contributions presented in the study are included in the article/**Supplementary Material**. Further inquiries can be directed to the corresponding authors.

## Ethics Statement

The studies involving human participants were reviewed and approved by the ethics committees of the First Affiliated Hospital of Sun Yat-sen University. Written informed consent for participation was not required for this study in accordance with the national legislation and the institutional requirements.

## Author Contributions

BL, LL, LW, YW, LC, QC, HX, and SC made substantial contributions to conception and design, acquisition of data, and analysis and interpretation of data. LL, HX, SC, SP, SL, and MK participated in drafting the article or revising it critically for important intellectual content. QZ assisted in making statistical analysis according to comments from reviewers. SL and MK gave final approval of the version to be published. All authors contributed to the article and approved the submitted version.

## Funding

The present work was supported by grants from National Key Research and Development Program of China (No. 2020AAA0109504) and the Guangdong Basic and Applied Basic Research Foundation (No. 2019A1515111168).

## Conflict of Interest

The authors declare that the research was conducted in the absence of any commercial or financial relationships that could be construed as a potential conflict of interest.

## Publisher’s Note

All claims expressed in this article are solely those of the authors and do not necessarily represent those of their affiliated organizations, or those of the publisher, the editors and the reviewers. Any product that may be evaluated in this article, or claim that may be made by its manufacturer, is not guaranteed or endorsed by the publisher.
