# Supplementary material for: Innovative Synoptic Reporting With Seven-Point Sampling Protocol to Improve Detection Rate of Microvascular Invasion in Hepatocellular Carcinoma
Source: Front Oncol. 2021 Nov 4;11:726239. doi: 10.3389/fonc.2021.726239 (PMC8599152; doi:10.3389/fonc.2021.726239)
Supplement: Supplementary file 1 [file Table_1.docx]

**Electronic Supplementary Material**

**S1 Table Comparisons of clinicopathological characteristics between MVI-positive and MVI-negative groups after SPRING in our center**

| Variables | Levels | Total | MVI- | MVI+ | Pvalue^1^ |
| --- | --- | --- | --- | --- | --- |
| Age(yr) | Mean(SD) | 53.7(11.1) | 55.3(10.6) | 52.1(11.4) | 0.005 |
|  | Median(IQR) | 53.4 (46.2,62.1) | 55.4 (47.8,62.7) | 51.5 (43.9,61.4) |  |
| Gender | Male | 335(87.7%) | 167(87.0%) | 168(88.4%) | 0.668 |
|  | Female | 47(12.3%) | 25(13.0%) | 22(11.6%) |  |
| HBsAg | Negative | 62(16.2%) | 33(17.2%) | 29(15.3%) | 0.610 |
|  | Positive | 320(83.8%) | 159(82.8%) | 161(84.7%) |  |
| HCV | Negative | 367(96.1%) | 186(96.9%) | 181(95.3%) | 0.417 |
|  | Positive | 15(3.9%) | 6(3.1%) | 9(4.7%) |  |
| PLT(×10^9^/L) | <100 | 26(6.8%) | 17(8.9%) | 9(4.7%) | 0.110 |
|  | ≥100 | 356(93.2%) | 175(91.1%) | 181(95.3%) |  |
| AFP level | Mean(SD) | 18224(107E3) | 3778(38317) | 32822(145E3) | <0.001# |
| (ng/mL) | Median(IQR) | 25.9 (4.9,577.7) | 10.4 (3.5,179.8) | 107.7 (9.2,1577) |  |
| AFP group | ≤20 | 179(46.9%) | 114(59.4%) | 65(34.2%) | <0.001 |
| (ng/mL) | 20-400 | 97(25.4%) | 44(22.9%) | 53(27.9%) |  |
|  | ≥400 | 106(27.7%) | 34(17.7%) | 72(37.9%) |  |
| Tumor size(cm) | Mean(SD) | 6.1(6.8) | 4.7(3.4) | 7.5(8.9) | <0.001# |
|  | Median(IQR) | 4.8 (3.3,7.1) | 4.0 (2.8,5.7) | 5.9 (4.1,8.6) |  |
|  | N(Min,Max) | 382(1.2,100.0) | 192(1.4,40.0) | 190(1.2,100.0) |  |
| Tumor size group | 1-3cm | 81(21.2%) | 56(29.2%) | 25(13.2%) | <0.001 |
|  | 3-5cm | 114(29.8%) | 70(36.5%) | 44(23.2%) |  |
|  | ≥5cm | 187(49.0%) | 66(34.4%) | 121(63.7%) |  |
| Tumor number | 1 | 304(79.6%) | 160(83.3%) | 144(75.8%) | 0.333 |
| group | 2 | 45(11.8%) | 19(9.9%) | 26(13.7%) |  |
|  | 3 | 10(2.6%) | 4(2.1%) | 6(3.2%) |  |
|  | >3 | 23(6.0%) | 9(4.7%) | 14(7.4%) |  |
| BCLC group | 0A | 314(82.2%) | 164(85.4%) | 150(78.9%) | 0.098 |
|  | B | 68(17.8%) | 28(14.6%) | 40(21.1%) |  |

1. Principles for selecting P values and statistics: (1) For continuous variables, if they met normal distribution, we used T-test results, otherwise we used Wilcoxon results ("#" means that continuous variables did not meet normal distribution). (2) For categorical variables, we used chi-square test or Fisher exact probability method. 2. Data description method: (1) For continuous variables, if they satisfied normal distribution, we selected the mean (standard deviation); otherwise, we selected the median (interquartile range IQR). (2) For categorical variables, they were described as N (%) under different categories.

**S2 Table Comparisons of clinicopathological characteristics between MVI-positive and MVI-negative groups in external centers (2017.7-2019.12)**

| Variables | Levels | Total | MVI- | MVI+ | Pvalue^1^ |
| --- | --- | --- | --- | --- | --- |
| Age(yr) | Mean(SD) | 56.5(12.3) | 56.7(12.6) | 55.2(10.6) | 0.417 |
|  | Median(IQR) | 57.1 (46.6,65.7) | 57.4 (46.6,66.1) | 53.2 (46.9,60.5) |  |
| Gender | Male | 99(86.8%) | 85(86.7%) | 14(87.5%) | 1.000 |
|  | Female | 15(13.2%) | 13(13.3%) | 2(12.5%) |  |
| HBsAg | Negative | 30(26.3%) | 25(25.5%) | 5(31.3%) | 0.760 |
|  | Positive | 84(73.7%) | 73(74.5%) | 11(68.8%) |  |
| HCV | Negative | 113(99.1%) | 97(99.0%) | 16(100.0%) | 1.000 |
|  | Positive | 1(0.9%) | 1(1.0%) | 0(0.0%) |  |
| PLT(×10^9^/L) | <100 | 11(9.6%) | 11(11.2%) | 0(0.0%) | 0.358 |
|  | ≥100 | 103(90.4%) | 87(88.8%) | 16(100.0%) |  |
| AFP level | Mean(SD) | 5972(24373) | 3220(10389) | 22828(58521) | 0.104# |
| (ng/mL) | Median(IQR) | 65.5 (5.2,1197) | 52.2 (4.4,1191) | 380.8 (58.5,2958) |  |
| AFP group | ≤20 | 45(39.5%) | 42(42.9%) | 3(18.8%) | 0.169 |
| (ng/mL) | 20-400 | 30(26.3%) | 25(25.5%) | 5(31.3%) |  |
|  | ≥400 | 39(34.2%) | 31(31.6%) | 8(50.0%) |  |
| Tumor size(cm) | Mean(SD) | 5.7(3.3) | 5.3(2.9) | 8.2(4.6) | 0.010# |
|  | Median(IQR) | 5.2 (3.3,7.1) | 4.8 (3.3,6.3) | 7.5 (5.2,11.4) |  |
| Tumor size group | 1-3cm | 25(21.9%) | 23(23.5%) | 2(12.5%) | 0.039 |
|  | 3-5cm | 30(26.3%) | 29(29.6%) | 1(6.3%) |  |
|  | ≥5cm | 59(51.8%) | 46(46.9%) | 13(81.3%) |  |
| Tumor number | 1 | 106(93.0%) | 93(94.9%) | 13(81.3%) | 0.091 |
| group | 2 | 6(5.3%) | 4(4.1%) | 2(12.5%) |  |
|  | >3 | 2(1.8%) | 1(1.0%) | 1(6.3%) |  |
| BCLC group | 0A | 106(93.0%) | 93(94.9%) | 13(81.3%) | 0.082 |
|  | B | 8(7.0%) | 5(5.1%) | 3(18.8%) |  |

1. Principles for selecting P values and statistics: (1) For continuous variables, if they met normal distribution, we used T-test results, otherwise we used Wilcoxon results ("#" means that continuous variables did not meet normal distribution). (2) For categorical variables, we used chi-square test or Fisher exact probability method. 2. Data description method: (1) For continuous variables, if they satisfied normal distribution, we selected the mean (standard deviation); otherwise, we selected the median (interquartile range IQR). (2) For categorical variables, they were described as N (%) under different categories.

**S3 Table Comparisons of baseline characters between retrospective and prospective cohort in our center**

| Variables | Levels | Total | 200903-201706 | 201707-201912 | Pvalue^1^ |
| --- | --- | --- | --- | --- | --- |
| Age(yr) | Mean(SD) | 53.8(11.5) | 53.9(11.7) | 53.7(11.1) | 0.612# |
|  | Median(IQR) | 54.4 (45.8,62.1) | 54.7 (45.5,62.1) | 53.4 (46.2,62.1) |  |
| Gender | Male | 998(86.8%) | 663(86.3%) | 335(87.7%) | 0.519 |
|  | Female | 152(13.2%) | 105(13.7%) | 47(12.3%) |  |
| HBsAg | Negative | 175(15.2%) | 113(14.7%) | 62(16.2%) | 0.500 |
|  | Positive | 975(84.8%) | 655(85.3%) | 320(83.8%) |  |
| HCV | Negative | 1117(97.1%) | 750(97.7%) | 367(96.1%) | 0.130 |
|  | Positive | 33(2.9%) | 18(2.3%) | 15(3.9%) |  |
| PLT(×10^9^/L) | <100 | 104(9.0%) | 78(10.2%) | 26(6.8%) | 0.062 |
|  | ≥100 | 1046(91.0%) | 690(89.8%) | 356(93.2%) |  |
| AFP level | Mean(SD) | 16631(11E4) | 15838(111E3) | 18224(107E3) | 0.095# |
| (ng/mL) | Median(IQR) | 43.7 (5.5,667.8) | 53.9 (5.9,756.1) | 25.9 (4.9,577.7) |  |
| AFP group | ≤20 | 483(42.0%) | 304(39.6%) | 179(46.9%) | 0.060 |
| (ng/mL) | 20-400 | 324(28.2%) | 227(29.6%) | 97(25.4%) |  |
|  | ≥400 | 343(29.8%) | 237(30.9%) | 106(27.7%) |  |
| Tumor size(cm) | Mean(SD) | 5.8(4.7) | 5.6(3.1) | 6.1(6.8) | 0.676# |
|  | Median(IQR) | 4.8 (3.4,7.3) | 4.8 (3.4,7.3) | 4.8 (3.3,7.1) |  |
| Tumor size group | 1-3cm | 222(19.3%) | 141(18.4%) | 81(21.2%) | 0.301 |
|  | 3-5cm | 374(32.5%) | 260(33.9%) | 114(29.8%) |  |
|  | ≥5cm | 554(48.2%) | 367(47.8%) | 187(49.0%) |  |
| Tumor number | 1 | 908(79.0%) | 604(78.6%) | 304(79.6%) | 0.889 |
| group | 2 | 132(11.5%) | 87(11.3%) | 45(11.8%) |  |
|  | 3 | 35(3.0%) | 25(3.3%) | 10(2.6%) |  |
|  | >3 | 75(6.5%) | 52(6.8%) | 23(6.0%) |  |
| BCLC group | 0A | 937(81.5%) | 623(81.1%) | 314(82.2%) | 0.657 |
|  | B | 213(18.5%) | 145(18.9%) | 68(17.8%) |  |

1. Principles for selecting P values and statistics: (1) For continuous variables, if they met normal distribution, we used T-test results, otherwise we used Wilcoxon results ("#" means that continuous variables did not meet normal distribution). (2) For categorical variables, we used chi-square test or Fisher exact probability method. 2. Data description method: (1) For continuous variables, if they satisfied normal distribution, we selected the mean (standard deviation); otherwise, we selected the median (interquartile range IQR). (2) For categorical variables, they were described as N (%) under different categories.

**S4 Table Comparisons of baseline characters between retrospective and prospective cohort in external centers**

| Variables | Levels | Total | Retrospective cohort | Prospective cohort | Pvalue^1^ |
| --- | --- | --- | --- | --- | --- |
| Age(yr) | Mean(SD) | 54.8(11.7) | 54.0(11.4) | 56.5(12.3) | 0.061 |
|  | Median(IQR) | 56.0 (45.8,63.0) | 55.0 (45.4,61.8) | 57.1 (46.6,65.7) |  |
| Gender | Male | 310(88.1%) | 211(88.7%) | 99(86.8%) | 0.623 |
|  | Female | 42(11.9%) | 27(11.3%) | 15(13.2%) |  |
| HBsAg | Negative | 61(17.3%) | 31(13.0%) | 30(26.3%) | 0.002 |
|  | Positive | 291(82.7%) | 207(87.0%) | 84(73.7%) |  |
| HCV | Negative | 347(98.6%) | 234(98.3%) | 113(99.1%) | 1.000 |
|  | Positive | 5(1.4%) | 4(1.7%) | 1(0.9%) |  |
| PLT(×10^9^/L) | <100 | 44(12.5%) | 33(13.9%) | 11(9.6%) | 0.263 |
|  | ≥100 | 308(87.5%) | 205(86.1%) | 103(90.4%) |  |
| AFP level | Mean(SD) | 10771(47289) | 13069(54884) | 5972(24373) | 0.558# |
| (ng/mL) | Median(IQR) | 39.0 (4.9,1183) | 31.8 (4.8,1092) | 65.5 (5.2,1197) |  |
| AFP group | ≤20 | 152(43.2%) | 107(45.0%) | 45(39.5%) | 0.620 |
| (ng/mL) | 20-400 | 88(25.0%) | 58(24.4%) | 30(26.3%) |  |
|  | ≥400 | 112(31.8%) | 73(30.7%) | 39(34.2%) |  |
| Tumor size(cm) | Mean(SD) | 5.8(3.6) | 5.9(3.7) | 5.7(3.3) | 0.671# |
|  | Median(IQR) | 5.2 (3.3,7.1) | 5.2 (3.3,7.0) | 5.2 (3.3,7.1) |  |
| Tumor size group | 1-3cm | 71(20.2%) | 46(19.3%) | 25(21.9%) | 0.782 |
|  | 3-5cm | 90(25.6%) | 60(25.2%) | 30(26.3%) |  |
|  | ≥5cm | 191(54.3%) | 132(55.5%) | 59(51.8%) |  |
| Tumor number | 1 | 311(88.4%) | 205(86.1%) | 106(93.0%) | 0.162 |
| group | 2 | 21(6.0%) | 15(6.3%) | 6(5.3%) |  |
|  | 3 | 2(0.6%) | 2(0.8%) | 0(0.0%) |  |
|  | >3 | 18(5.1%) | 16(6.7%) | 2(1.8%) |  |
| BCLC group | 0A | 313(88.9%) | 207(87.0%) | 106(93.0%) | 0.093 |
|  | B | 39(11.1%) | 31(13.0%) | 8(7.0%) |  |

1. Principles for selecting P values and statistics: (1) For continuous variables, if they met normal distribution, we used T-test results, otherwise we used Wilcoxon results ("#" means that continuous variables did not meet normal distribution). (2) For categorical variables, we used chi-square test or Fisher exact probability method. 2. Data description method: (1) For continuous variables, if they satisfied normal distribution, we selected the mean (standard deviation); otherwise, we selected the median (interquartile range IQR). (2) For categorical variables, they were described as N (%) under different categories.

**S5 Table Comparisons of baseline characters between our center and external centers**

| Variables | Levels | Total | Our Center | External Centers | Pvalue^1^ |
| --- | --- | --- | --- | --- | --- |
| Age(yr) | Mean(SD) | 54.1(11.6) | 53.8(11.5) | 54.8(11.7) | 0.206# |
|  | Median(IQR) | 54.7 (45.8,62.4) | 54.4 (45.8,62.1) | 56.0 (45.8,63.0) |  |
| Gender | Male | 1308(87.1%) | 998(86.8%) | 310(88.1%) | 0.529 |
|  | Female | 194(12.9%) | 152(13.2%) | 42(11.9%) |  |
| HBsAg | Negative | 236(15.7%) | 175(15.2%) | 61(17.3%) | 0.341 |
|  | Positive | 1266(84.3%) | 975(84.8%) | 291(82.7%) |  |
| HCV | Negative | 1464(97.5%) | 1117(97.1%) | 347(98.6%) | 0.130 |
|  | Positive | 38(2.5%) | 33(2.9%) | 5(1.4%) |  |
| PLT(×10^9^/L) | <100 | 148(9.9%) | 104(9.0%) | 44(12.5%) | 0.057 |
|  | ≥100 | 1354(90.1%) | 1046(91.0%) | 308(87.5%) |  |
| AFP level | Mean(SD) | 15257(98637) | 16631(11E4) | 10771(47289) | 0.897# |
| (ng/mL) | Median(IQR) | 41.6 (5.3,744.7) | 43.7 (5.5,667.8) | 39.0 (4.9,1183) |  |
| AFP group | ≤20 | 635(42.3%) | 483(42.0%) | 152(43.2%) | 0.489 |
| (ng/mL) | 20-400 | 412(27.4%) | 324(28.2%) | 88(25.0%) |  |
|  | ≥400 | 455(30.3%) | 343(29.8%) | 112(31.8%) |  |
| Tumor size(cm) | Mean(SD) | 5.8(4.4) | 5.8(4.7) | 5.8(3.6) | 0.527# |
|  | Median(IQR) | 4.9 (3.4,7.2) | 4.8 (3.4,7.3) | 5.2 (3.3,7.1) |  |
| Tumor size group | 1-3cm | 293(19.5%) | 222(19.3%) | 71(20.2%) | 0.042 |
|  | 3-5cm | 464(30.9%) | 374(32.5%) | 90(25.6%) |  |
|  | ≥5cm | 745(49.6%) | 554(48.2%) | 191(54.3%) |  |
| Tumor number | 1 | 1219(81.2%) | 908(79.0%) | 311(88.4%) | <0.001 |
| group | 2 | 153(10.2%) | 132(11.5%) | 21(6.0%) |  |
|  | 3 | 37(2.5%) | 35(3.0%) | 2(0.6%) |  |
|  | >3 | 93(6.2%) | 75(6.5%) | 18(5.1%) |  |
| BCLC group | 0A | 1250(83.2%) | 937(81.5%) | 313(88.9%) | 0.001 |
|  | B | 252(16.8%) | 213(18.5%) | 39(11.1%) |  |

1. Principles for selecting P values and statistics: (1) For continuous variables, if they met normal distribution, we used T-test results, otherwise we used Wilcoxon results ("#" means that continuous variables did not meet normal distribution). (2) For categorical variables, we used chi-square test or Fisher exact probability method. 2. Data description method: (1) For continuous variables, if they satisfied normal distribution, we selected the mean (standard deviation); otherwise, we selected the median (interquartile range IQR). (2) For categorical variables, they were described as N (%) under different categories.

**S6 Table Comparisons of baseline characters between 2018 and 2019 in our center**

| Variables | Levels | Total | 2018 | 2019 | Pvalue^1^ |
| --- | --- | --- | --- | --- | --- |
| Age(yr) | Mean(SD) | 54.6(11.0) | 55.0(10.9) | 54.2(11.1) | 0.484 |
|  | Median(IQR) | 54.1 (46.9,62.8) | 54.5 (47.1,63.7) | 53.9 (46.7,62.6) |  |
| Gender | Male | 284(87.7%) | 140(86.4%) | 144(88.9%) | 0.499 |
|  | Female | 40(12.3%) | 22(13.6%) | 18(11.1%) |  |
| HBsAg | Negative | 50(15.4%) | 27(16.7%) | 23(14.2%) | 0.538 |
|  | Positive | 274(84.6%) | 135(83.3%) | 139(85.8%) |  |
| HCV | Negative | 312(96.3%) | 157(96.9%) | 155(95.7%) | 0.556 |
|  | Positive | 12(3.7%) | 5(3.1%) | 7(4.3%) |  |
| PLT(×10^9^/L) | <100 | 24(7.4%) | 11(6.8%) | 13(8.0%) | 0.671 |
|  | ≥100 | 300(92.6%) | 151(93.2%) | 149(92.0%) |  |
| AFP level | Mean(SD) | 20597(116E3) | 26013(126E3) | 15180(104E3) | 0.683# |
| (ng/mL) | Median(IQR) | 27.8 (5.0,526.6) | 18.2 (4.9,678.8) | 38.5 (5.0,413.5) |  |
| AFP group | ≤20 | 151(46.6%) | 83(51.2%) | 68(42.0%) | 0.014 |
| (ng/mL) | 20-400 | 83(25.6%) | 30(18.5%) | 53(32.7%) |  |
|  | ≥400 | 90(27.8%) | 49(30.2%) | 41(25.3%) |  |
| Tumor size(cm) | Mean(SD) | 6.2(7.3) | 6.8(9.0) | 5.5(5.1) | 0.001# |
|  | Median(IQR) | 5.0 (3.3,7.1) | 5.6 (3.8,8.0) | 4.4 (2.8,6.5) |  |
| Tumor size group | 1-3cm | 70(21.6%) | 22(13.6%) | 48(29.6%) | 0.001 |
|  | 3-5cm | 92(28.4%) | 46(28.4%) | 46(28.4%) |  |
|  | ≥5cm | 162(50.0%) | 94(58.0%) | 68(42.0%) |  |
| Tumor number | 1 | 256(79.0%) | 125(77.2%) | 131(80.9%) | 0.796 |
| group | 2 | 38(11.7%) | 21(13.0%) | 17(10.5%) |  |
|  | 3 | 8(2.5%) | 5(3.1%) | 3(1.9%) |  |
|  | >3 | 22(6.8%) | 11(6.8%) | 11(6.8%) |  |
| BCLC group | 0A | 263(81.2%) | 128(79.0%) | 135(83.3%) | 0.320 |
|  | B | 61(18.8%) | 34(21.0%) | 27(16.7%) |  |
| MVI rate | MVI- | 164(50.6%) | 75(46.3%) | 89(54.9%) | 0.120 |
|  | MVI+ | 160(49.4%) | 87(53.7%) | 73(45.1%) |  |

1. Principles for selecting P values and statistics: (1) For continuous variables, if they met normal distribution, we used T-test results, otherwise we used Wilcoxon results ("#" means that continuous variables did not meet normal distribution). (2) For categorical variables, we used chi-square test or Fisher exact probability method. 2. Data description method: (1) For continuous variables, if they satisfied normal distribution, we selected the mean (standard deviation); otherwise, we selected the median (interquartile range IQR). (2) For categorical variables, they were described as N (%) under different categories.

**S7 Table Comparisons of baseline characters between 2018 and 2019 in external centers**

| Variables | Levels | Total | 2018 | 2019 | Pvalue^1^ |
| --- | --- | --- | --- | --- | --- |
| Age(yr) | Mean(SD) | 57.2(11.3) | 55.6(10.5) | 58.8(11.9) | 0.181 |
|  | Median(IQR) | 57.3 (47.6,66.1) | 55.8 (47.2,63.4) | 61.0 (49.7,66.2) |  |
| Gender | Male | 80(87.9%) | 40(88.9%) | 40(87.0%) | 0.777 |
|  | Female | 11(12.1%) | 5(11.1%) | 6(13.0%) |  |
| HBsAg | Negative | 23(25.3%) | 11(24.4%) | 12(26.1%) | 0.857 |
|  | Positive | 68(74.7%) | 34(75.6%) | 34(73.9%) |  |
| HCV | Negative | 90(98.9%) | 45(100.0%) | 45(97.8%) | 1.000 |
|  | Positive | 1(1.1%) | 0(0.0%) | 1(2.2%) |  |
| PLT(×10^9^/L) | <100 | 10(11.0%) | 4(8.9%) | 6(13.0%) | 0.739 |
|  | ≥100 | 81(89.0%) | 41(91.1%) | 40(87.0%) |  |
| AFP level | Mean(SD) | 3480(17854) | 1765(5256) | 5158(24590) | 0.329# |
| (ng/mL) | Median(IQR) | 58.4 (5.2,677.7) | 122.2 (8.7,478.0) | 30.2 (4.0,1304) |  |
| AFP group | ≤20 | 36(39.6%) | 14(31.1%) | 22(47.8%) | 0.203 |
| (ng/mL) | 20-400 | 26(28.6%) | 16(35.6%) | 10(21.7%) |  |
|  | ≥400 | 29(31.9%) | 15(33.3%) | 14(30.4%) |  |
| Tumor size(cm) | Mean(SD) | 5.7(3.3) | 6.1(3.7) | 5.3(2.8) | 0.407# |
|  | Median(IQR) | 5.0 (3.6,7.1) | 5.2 (3.8,7.6) | 4.8 (3.6,6.0) |  |
| Tumor size group | 1-3cm | 19(20.9%) | 10(22.2%) | 9(19.6%) | 0.412 |
|  | 3-5cm | 26(28.6%) | 10(22.2%) | 16(34.8%) |  |
|  | ≥5cm | 46(50.5%) | 25(55.6%) | 21(45.7%) |  |
| Tumor number | 1 | 83(91.2%) | 38(84.4%) | 45(97.8%) | 0.045 |
| group | 2 | 6(6.6%) | 5(11.1%) | 1(2.2%) |  |
|  | >3 | 2(2.2%) | 2(4.4%) | 0(0.0%) |  |
| BCLC group | 0A | 83(91.2%) | 38(84.4%) | 45(97.8%) | 0.030 |
|  | B | 8(8.8%) | 7(15.6%) | 1(2.2%) |  |
| MVI rate | MVI- | 79(86.8%) | 35(77.8%) | 44(95.7%) | 0.012 |
|  | MVI+ | 12(13.2%) | 10(22.2%) | 2(4.3%) |  |

1.Principles for selecting P values and statistics: (1) For continuous variables, if they met normal distribution, we used T-test results, otherwise we used Wilcoxon results ("#" means that continuous variables did not meet normal distribution). (2) For categorical variables, we used chi-square test or Fisher exact probability method. 2. Data description method: (1) For continuous variables, if they satisfied normal distribution, we selected the mean (standard deviation); otherwise, we selected the median (interquartile range IQR). (2) For categorical variables, they were described as N (%) under different categories.

**A Listing of Supplemental Digital Content**

S1 Table Comparisons of clinicopathological characteristics between MVI-positive and MVI-negative groups after SPRING in our center

S2 Table Comparisons of clinicopathological characteristics between MVI-positive and MVI-negative groups after SPRING in external centers

S3 Table Comparisons of baseline characters between retrospective and prospective cohort in our center

S4 Table Comparisons of baseline characters between retrospective and prospective cohort in external centers

S5 Table Comparisons of baseline characters between our center and external centers

S6 Table Comparisons of baseline characters between 2018 and 2019 in our center

S7 Table Comparisons of baseline characters between 2018 and 2019 in external centers
